# Dental Healthcare Amid the COVID-19 Pandemic

**DOI:** 10.3390/ijerph182111008

**Published:** 2021-10-20

**Authors:** Rabia Tariq Butt, Omer Sefvan Janjua, Sana Mehmood Qureshi, Muhammad Saad Shaikh, Julia Guerrero-Gironés, Francisco J. Rodríguez-Lozano, Muhammad Sohail Zafar

**Affiliations:** 1 General Dental Practitioner, Al-Noor Clinics, Okara 56000, Pakistan; rabiiiatariq@gmail.com; 2 Department of Maxillofacial Surgery, PMC Dental Institute, Faisalabad Medical University, Faisalabad 38000, Pakistan; osj1982@hotmail.com; 3 Department of Oral Pathology, PMC Dental Institute, Faisalabad Medical University, Faisalabad 38000, Pakistan; sana.mehmood@outlook.com; 4 Department of Oral Biology, Sindh Institute of Oral Health Sciences, Jinnah Sindh Medical University, Karachi 75510, Pakistan; drsaadtanvir@gmail.com; 5 Gerodontology and Special Care Dentistry Unit, Hospital Morales Meseguer, Medicine School, University of Murcia, 30100 Murcia, Spain; fcojavier@um.es; 6 Cellular Therapy and Hematopoietic Transplant Research Group, Biomedical Research Institute of Murcia, Clinical University Hospital Virgen de laArrixaca, University of Murcia, 30120 Murcia, Spain; 7 Department of Restorative Dentistry, College of Dentistry, Taibah University, Al Madinah, Al Munawwarah 41311, Saudi Arabia; MZAFAR@taibahu.edu.sa or; 8 Department of Dental Materials, Islamic International Dental College, Riphah International University, Islamabad 44000, Pakistan

**Keywords:** SARS-CoV-2, dentistry, dental treatment, coronavirus

## Abstract

The hustle and bustle of the planet Earth have come to a halt thanks to the novel coronavirus. The virus has affected approximately 219 million people globally; taken the lives of 4.55 million patients as of September 2021; and created an ambiance of fear, social distancing, and economic instability. The purpose of this review article is to trace the historical origin and evolution of severe acute respiratory syndrome coronavirus type 2 (SARS-CoV-2). The virus is highly contagious with a unique feature of rapid mutations—the scientific research is paving the way for discoveries regarding novel coronavirus disease (COVID-19) diagnosis, features, prevention, and vaccination. The connections between the coronavirus pandemic and dental practices are essential because COVID-19 is transmitted by aerosols, fomites, and respiratory droplets, which are also produced during dental procedures, putting both the patient and the dentist at risk. The main emphasis of this paper is to highlight the psychological, economic, and social impact of this pandemic on dental practices throughout the world and under what circumstances and guidelines can dental health care be provided. In the current situation of the pandemic, an appropriate screening tool must be established either by using rapid molecular testing or saliva point-of-care technology, which will be effective in identifying as well as isolating the potential contacts and carriers in hopes to contain and mitigate infection. The blessing in disguise is that this virus has united the leaders, scientists, health care providers, and people of all professions from all around the world to fight against a common enemy.

## 1. Introduction

Coronaviruses are a group of viruses causing common cold and flu-like symptoms, infecting both humans and animals [1]. Novel coronavirus disease (COVID-19) is an infectious disorder [1] due to severe acute respiratory syndrome caused by coronavirus type 2 (SARS-CoV-2). The first diagnosed case was in the “wet markets’’ of Wuhan (China) in December 2019 [1]. On 11 March 2020, the disease was announced as a global pandemic [2] by the World Health Organization (WHO). The WHO reports more than 219 million COVID-19 cases globally [3]. SARS-CoV-2 is 88% identical to severe acute respiratory syndrome (SARS-like CoV) derived from bats collected from eastern China in 2018 [4]. It possesses 79% genetic resemblance with SARS-CoV (2003) and 50% with Middle East respiratory syndrome coronavirus (MERS-CoV) [4]. This virus is highly temperature-sensitive with maximum stability at 4 °C, but when the temperature is raised to 70 °C during the incubation period its inactivation time is reduced to 5 min [5,6]. Novel coronavirus (2019-nCoV) can remain stable on inanimate surfaces up to 1.5 week, up to 180 min on printing papers, up to 48 h on clothes and wood, up to 96 h on smooth surfaces such as glass, and may survive for several days on stainless steel, plastics, and the inner or outer surface of surgical masks [5,7]. The purpose of the present review is to emphasize the impact of COVID-19 on dentistry, how the dentist can aid in its diagnosis, and its overall effects on the profession of dentistry worldwide. In addition to this, the article also highlights the role of teledentistry and newly established guidelines for providing dental health care amid the pandemic.

## 2. History and Evolution Novel Coronavirus

Epidemiological and genetic studies show that SARS-CoV-2 is zoonotic in origin [8]. The first transmission was from animal (bats and pangolins) to human (zoonosis), followed by inter-human transmissions [9]. However, human–animal transmission (anthroponosis) has also been hypothesized [10]. For instance, SARS-CoV-2 infected minks were reported in Sweden, Denmark, United States, Spain, and Netherlands [11]. Domestic dogs and cats of infected persons have also tested positive for SARS-CoV-2 [12]. Dr. Almeida first identified coronavirus at St. Thomas Hospital, London, in 1964, where the virus caused flu-like symptoms in humans [13]. By 1967, scientists discovered similar human and animal viruses and called them due to their crown-like appearances [13]. In 2002, SARS-CoV emerged in southern China, spreading to 28 other countries [14]. More than 8000 people were infected by July 2003, with a mortality rate of 10% [14]. In 2012, MERS-CoV affected 1700 people in Saudi Arabia with a mortality rate of 36% [15,16]. The 2019-nCoV infecting humans was identified with the aid of next-generation sequencing by the end of 2019 [17]. Since its emergence in 2019, SARS-CoV-2 has shown genetic diversity, attributed to a low fidelity viral polymerase and increased recombination frequency. Both of these factors promote a high mutation rate. These genomic mutations of COVID-19 virus have generated variants which have been sorted out in nine clades viz L, V, S, G, GH, GR, GV, GRY, and O. At the time of writing, clades G, GH, GR, and GRY are responsible for the majority of infections while clades L and V, which were responsible for the commencement of pandemic in December 2019, are almost extinct now. Since January 2021 onwards, B.1.1.7, B.1.351, P.1, and the Delta variant (B.1.617.2) derivatives of clade G are the predominant infections. These variants are demonstrating D614G, P323L, and F106F mutations, thus increasing the susceptibility of the virus for dissemination of disease [18]. D614G mutation first appeared in March 2020; B.1.1.7, which was labelled as UK variant, first emerged in September 2020. The South African variant (B.1.351) was first identified in December 2020 while the Brazilian variant or P.1 was first isolated in January 2021. B.1.617.2 was called the double variant and was first identified in December 2020 [19].

## 3. Specific Virology, Pathophysiology, and Life Cycle of COVID-19

Coronaviridae family possess a single-stranded RNA with the genomic length ranging from 26 to 32 kilobases [1] and can adapt to new environments through mutations causing long-term health effects [20]. The virus demonstrates round, elliptical, or a pleomorphic shape with an approximate diameter of 60–140 nm.

**Figure 1:**
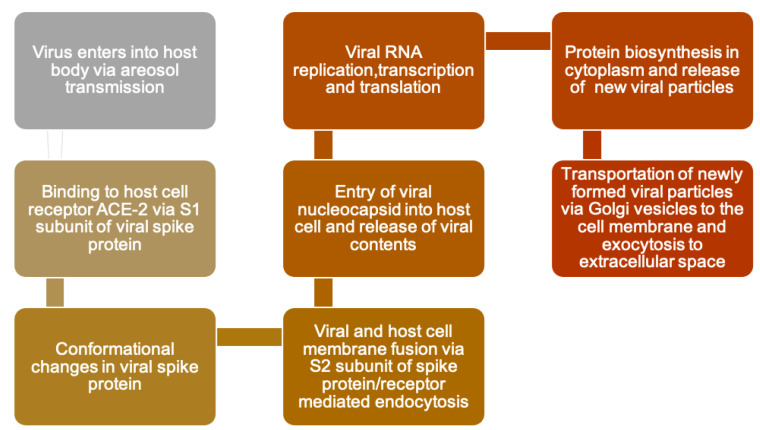
SARS-CoV-2 life cycle showing virus behaviour inside the body [21].

**Figure 2:**
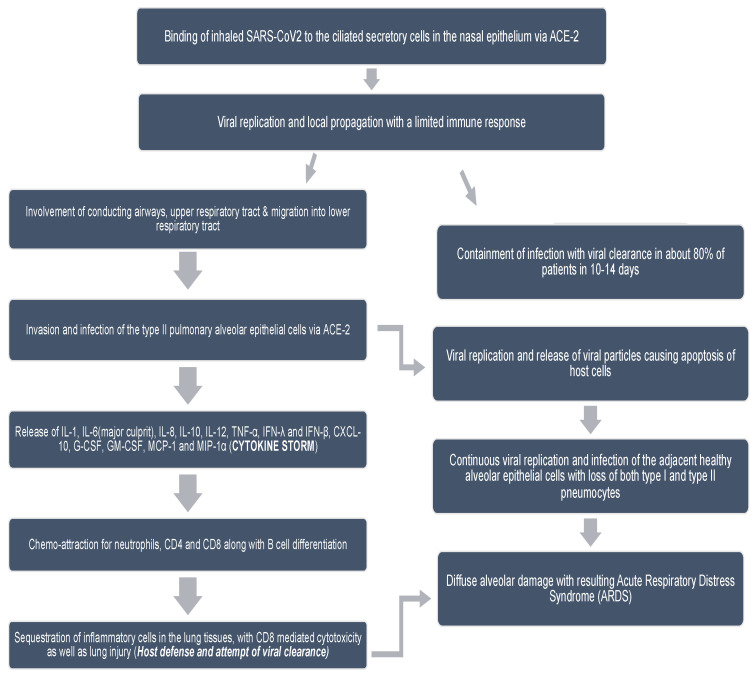
COVID-19 pathophysiology demonstrating disease progression from virus entrance in the body to causing acute respiratory distress syndrome (ARDS) [21].

The SARS-CoV-2 life cycle [21], once entered into the human body, is presented in Figure 1. The SARS-CoV-2 pathophysiology, once it has entered the human body through aerosol, is presented in Figure 2. The flow chart describes how the virus replicates inside the human body and how the body reacts to the replication of the virus. Clinical characteristics of COVID-19 are given in Table 1 [22,23,24,25,26]. In serologic studies, 30% of individuals were symptomless [27]. However, the symptomatic patients reported coryza [27]. The re-infection frequency observed with SARS-CoV-2 is high and implies that antibody IgG is not protective [27].

The virus can infect individuals from all ages irrespective of any gender predilection; however, individuals who are above 60 years of age or those possessing co-morbidities like diabetes, asthma, obesity, ischemic heart disease, cancer, or patients who have undergone organ transplant comprise the high-risk group with almost a 12 times greater chance of fatality with SARS-CoV-2 infection than individuals falling in low risk category [21]. According to a study conducted in Jinyintan and Wuhan pulmonary hospital on 191 adult patients, 48% had comorbid conditions, including high blood pressure (30%), diabetes mellitus (19%), and coronary artery disease (8%) [28]. The New York State Department of Health (NYSDOH) conducted a survey where they analysed the relationship of co-morbidities associated with fatality and the results are presented in Table 2. Similarly, data from the Centre for Disease Control and Prevention (CDC) has shown that Blacks, Hispanics, and Asians are at an increased risk of contracting SARS-CoV-2 infection [21].

## 4. Transmission

**Figure 3:**
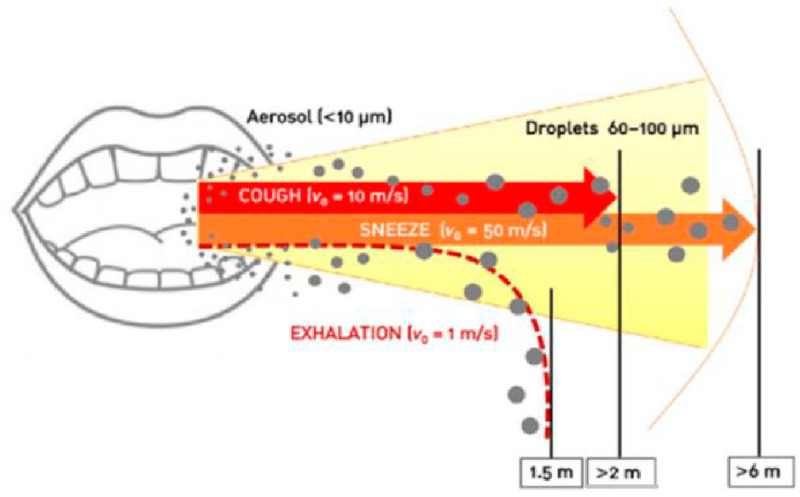
Showing exhalation distances of aerosols and droplets (Reprinted with permission from Ref. [31]. Copyright 2021 Elsevier.

The most common mode of human–human transmission occurs via droplets (coughing, sneezing, talking, and aerosol generating procedures) or blood with smaller droplets traveling a longer distance and larger droplets limited to nearby objects [30]. The effect of droplet size on the distance is shown in Figure 3.

Furthermore, the studies of conjunctival samples from suspected as well as confirmed 2019-nCoV cases advocates that exposure of eyes may be an efficient way for the virus entrance [32,33]; therefore, there is a need for using protective eye-wear while working in a dental practice and other areas where one may come in contact with potential and confirmed cases.

## 5. Diagnosis and Diagnostic Methods

The CDC, China, reported the viral genome sequence in the international database banks GenBank and Global Initiative on Sharing All Influenza Data [32,34]. It helped labs to develop a particular real-time polymerase chain reaction (RT-PCR) evaluation for its diagnosis [32]. Other diagnostic tests include molecular tests (digital PCR, next-generation sequencing, microarray analysis, and isothermal nucleic acid amplification) and antibody testing for prior infection [35]. The methods for testing are:

1. Nasopharyngeal swab.
2. Oropharyngeal swab.
3. Blood sampling for antibody detection (immunization).
4. Expectorated sputum in severe respiratory disease [36].

In terms of sensitivity and specificity of diagnostic methods, sensitivity is the test’s capacity to identify all infected individuals. In contrast, specificity is the test’s ability to detect a particular pathogen [37]. Sensitivity of the RT-PCR varied depending on the specimen type: pharyngeal swab (32%), nasal swab (63%), sputum (72–75%), and bronchoalveolar lavage (93–95%) [38].

### 5.1. Saliva as a Diagnostic Tool

COVID-19 has also been detected in the saliva of infected patients [33]. There are three suspected pathways for SARS-CoV to be present in saliva:

1. Lower and upper respiratory tract → exchange of liquid droplets between the oral cavity and respiratory tract → virus in saliva [8,36]
2. Blood → virus via gingival crevicular fluid (GCF) enters oral cavity→ saliva [39]
3. SARS-CoV-2 → infection of salivary glands (rhesus macaques) → saliva [40]

Saliva is a cheap, accessible, and non-invasive diagnostic method with minimal risk of transmission and is currently serving as a biomarker for diagnosing and screening different diseases [41,42], including viral, fungal, or bacterial infections; various types of cancers; cardiovascular diseases; and developmental as well as genetic diseases [43,44,45]. It has substantial biomarkers and components, including DNA and RNA, various microorganisms, immunoglobulins, and metabolites [46]. Salivary glands are also a reservoir for the angiotensin-converting enzyme 2 (ACE-2) receptor expression, the functional receptor for this virus [47,48]. Considerably lower expression of ACE-2 receptors has been found in the pharyngeal cells compared to the lower respiratory tract and salivary glands [47].

### 5.2. Gingival Crevicular Fluid

The GCF is an inflammatory exudate or serum transudate of the pathological or healthy periodontal tissues [49]. It is used for the detection of periodontal diseases, presence of drugs in the periodontal pockets through systematic circulation, and proteomic analysis [50] for isolation and assessment of different viruses (Herpes simplex, Epstein–Barr, and Cytomegalovirus) [51]. GCF can be collected by absorption technique using paper strips/points and can be a non-invasive method for isolating and diagnosing coronavirus and its pathway for entry into the oral cavity [52].

## 6. SARS-COV-2 Incubation Period in Humans

The mean incubation period after being infected is 5.1 days. During this period, the patient remains asymptomatic. Three weeks is the crucial period for COVID-19 because patients either died after 15 to 22 days or were discharged between 18 to 25 days from the onset of symptoms (Table 3) [28].

## 7. Management of COVID-19

At present, there are no approved and specific therapies for 2019-nCoV. Many immunotherapies and antiviral drugs are under investigation for COVID-19 (Table 4) [53].

Vaccination is considered the most effective defence against infectious diseases. The same is true for COVID-19; therefore, over 214 candidate vaccines from different pharmaceutical companies are being developed. The vaccines are broadly classified as: vaccines based on full-length S-protein, protein (RBD-based or S2 based subunit vaccines), inactivated vaccines, live attenuated vaccine, nucleic acid (DNA/mRNA) based vaccines, replicating and non-replicating viral vectors vaccine, and viral-like particle vaccine [69]. COVID-19 vaccines showed promising results by producing specific T cell-mediated immune responses and increased the number of neutralizing antibodies (NAbs) [56]. Different vaccines for COVID-19 available are demonstrated in Table 5. Vaccination priorities include:

(1) Healthcare professionals and inhabitants of long-term care facilities.
(2) Essential workers (such as transportation, food service, finance, and health) and individuals aged 75 years or older.
(3) Individuals aged 65 to 74 years; individuals aged 16 to 64 years with systemic conditions.

Once the people with priority have been successfully vaccinated, only then the general public will get the opportunity of getting vaccinated [70].

Vaccinations other than parenteral routes are also under development. Following are the examples of some of the vaccines which are under development and do not employ the parenteral route:

1. hAd5 T-cell (Immunity Bio and NantKwest) [76].
2. Intranasal COVID-19 vaccine (Ad COVID) [77].
3. ChAdOx1 nCov-19 inhaled (University of Oxford) [78].

Similarly, already available vaccines like BCG and MMR are being repurposed for developing immunity against COVID-19 as the literature has suggested that it may offer partial immunity against COVID-19 infection [71].

## 8. Oral Manifestations of COVID-19 Infection

**Figure 4:**
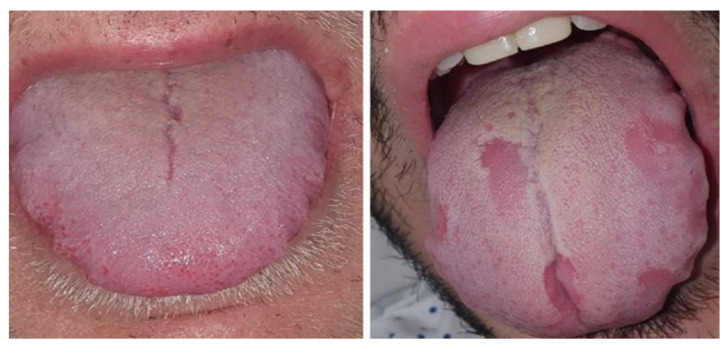
Macroglossia with lateral indentations (**left**) geographic tongue (**right**) (Reprinted with permission from Ref. [79]. Copyright 2020 John Wiley and Sons.

Evaluation of 666 patients at a temporary field hospital in Spain showed that 45% had mucocutaneous symptoms and more than 25% had oral symptoms as follows [79]: The relative frequencies of oral manifestations are 11.5% (lingual papillitis), 6.9% (aphthous stomatitis), 6.6% (glossitis), 5.3% (burning mouth sensation), and 3.9% (patchy depapillation and mucositis) [79]. Other common findings were macroglossia, tongue discoloration/coated tongue [80], and COVID-19 tongue (geographic tongue) (Figure 4) [81,82]. These oral lesions were left undiagnosed in COVID-19 patients because the patients usually wear masks while presenting in hospitals.

Other authors have reported association of COVID-19 with irregular and aphthous like lesions; herpetiform or zosteriform lesions; generalized non-specific ulcerations; erosions on the tongue, palate, and labial mucosa; atrophic and hyperkeratotic patches on the tongue, gingiva, and palate; lesions resembling erythema multiforme; desquamative gingivitis; and angina bullosa-like lesions [83]. Similarly, petechiae, post-inflammatory pigmentation, and vesicular eruptions have also been described. Periodontal manifestations include aggressive necrotizing periodontal disease, which may be due to bacterial co-infection caused by *Prevotella intermedia*. Two cases with Kawasaki disease and Melkerson–Rosenthal syndrome have also been reported [84]. Halepas et al. has reported oral manifestations of COVID-19 infection in paediatric patients. According to him, red swollen lips were seen in 48.9% cases while 10.6% presented with strawberry tongue and represent multi-system inflammatory syndrome in children [85].

In addition, SARS-CoV-2 is known to affect the salivary glands directly, which can lead to xerostomia and inflammation of the major salivary glands [86]. Dryness of the oral cavity can also be produced by mouth breathing, dehydration, and COVID-19 related medications. Severe halitosis has also been reported in COVID-19 patients [83]. Usually, this xerostomia is self-limiting and transient in nature. However, it can lead to periodontal disease and caries in patients run a protracted course of the disease or who are hospitalized for longer durations. Transneuronal migration of SARS-CoV-2 can lead to neuronal death of cells of the olfactory bulb and the taste buds, which can explain the loss of taste and smell [87]. Release of cytokines as in cytokine storm and acute febrile illness can lead to specific viral and non-specific ulcerations, fissuring, and erythematous eruptions in the oral cavity. The common areas of involvement include lips, tongues, palate, and buccal mucosa [88].

### 8.1. The Role of ACE-2 in Oral Manifestations of COVID-19

**Figure 5:**
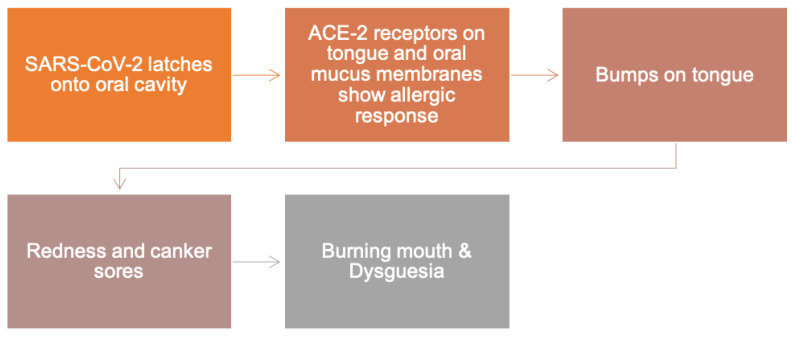
SARS-CoV-2 mechanism of oral manifestations. Reprinted with permission from Ref. [92]. Copyright 2020 John Wiley and Sons.

The ACE-2 is most likely to be the cell receptor of the 2019-nCoV [80]. The ACE-2 receptors which bind the SARS-CoV-2 are abundantly found on the surfaces of the oral mucosa, particularly the tongue and masticatory mucosa of the gingiva (Figure 5) [49,80]. These findings elucidate the risk for potentially substantial COVID-19 infectious susceptibility for the oral cavity and dental practices [49,80]. The strong affinity between ACE-2 and COVID-19 S protein proposed that the patients with greater ACE-2 expression are more liable to COVID-19 [89,90]. The cellular serine protease Transmembrane protease, serine 2 also added to the S-protein priming of 2019-nCoV, signifying its potential to comprise a management option [91].

### 8.2. Dentistry Hazards

The highest level of aerosol contamination within 60 cm between the patient’s head and dentist’s right arm has been shown in the literature. Aerosols can remain suspended in the air for 30 min after a dental procedure [93]. SARS-CoV-2 viability is estimated to be 3 h with an adherence capability for one and a half weeks to different surfaces [94].

## 9. Economic and Emotional Impact on Dentists and Dental Practices

**Figure 6:**
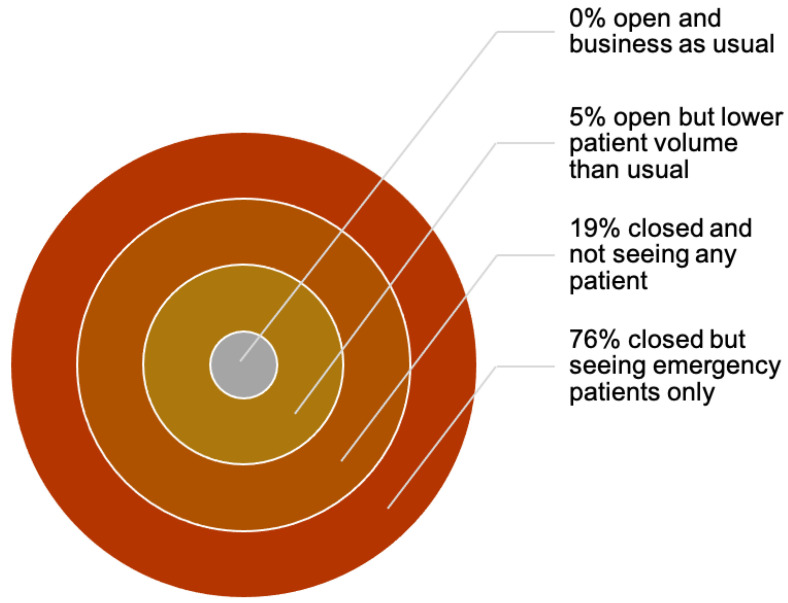
Economic impact of COVID-19 on dental practices [95].

For almost a year now, this pandemic has affected dental care providers both psychologically and economically (Figure 6) [95]. In various countries, dental procedures were banned entirely to prevent viral transmission, thus creating a huge financial implication for dental professionals.

The infection control during dental healthcare amid the COVID-19 pandemic is demonstrated in Table 6 [96,97,98,99,100,101]. In terms of the psychological impact of COVID-19 on dental personals, Khanagar et al. reported increased mental stress and psychological distress among dentists [102]. Depression, anxiety, fear, and stress have adversely affected dentists across the globe. They experienced fear of being exposed at the workplace and then the possibility of transmitting the infection to the near and dear ones at home [103]. The same was the case with dental students. Suspension of regular classes and closure of their schools took a toll on them as well. They felt anxious and depressed as they believed that they could not learn the skill during online teaching, which will affect their professional career. A study by Hakami et al. has reported that this anxiety and stress was more prevalent among female dentists and dental assistants as compared to their male counterparts [104]. Quarantines, isolations, financial impact, and loss of family members due to COVID-19 put immense pressures on dental surgeons and up to the extent that some dental personnel have expressed suicidal thoughts [105].

A study conducted by Ahmadi et al. in Iran concluded that around 10% of the dentists and their staff members had COVID-19 related symptoms. They have also reported that 63% of the dentists had faced financial problems due to COVID-19 pandemic and 43% reported anxiety and depression with almost 50% of these required consultation with a psychiatrist [106]. Kamran et al. conducted a nationwide survey among dentists in Pakistan and have reported that a significant number of dental practitioners have modified their practices following COVID-19 related guidelines. According to their survey around 70% dentists have installed a physical barrier at their workplace and have tried to maintain 6 feet distance in the waiting area. They also report that 70–80% dentists were using N95 masks and PPE like face shields, gowns, etc., and were avoiding aerosol generating procedures [107]. These studies clearly highlight the economical and emotional toll COVID-19 has on the dental profession.

Disinfectants containing 1000 mg/Chlorine for the walls, floors, and dental operatory disinfection. Alcohol-based sanitizer (75% to 80%) is beneficial against SARS-CoV-2. The suggested disinfectant for SARS-CoV-2 for waste disinfection before disposal is sodium hypochlorite [99]. Povidone-iodine (0.23% to 7%) and hydrogen peroxide (1.5%) are suggested to decrease viral load as a pre-procedural mouth wash [121,122]. Surface disinfectants including ethanol (62–71%), hydrogen peroxide (0.5%), and sodium hypochlorite (0.1%) are also efficient against SARS-CoV-2 [123,124].

## 10. Categorization of Dental Procedures according to COVID-19 Guidelines

Various dental procedures are categorized as emergencies and non-emergencies according to COVID-19 guidelines. Their details are presented in Table 7 below.

The transmission can be manageable, i.e., decrease the viral transmission (via close contact or droplets), using the point-of-care technology [126]. Several guidelines have been recommended to prevent and control the disease at various levels of populations. The WHO also advocated recommendations for the decrease in viral load via the disinfection and cleaning [127,128,129]:

1. Wash hands with alcohol-based soap solution for 20 s.
2. Wear masks when outside.
3. Avoid touching face.
4. Stay 6 feet apart from each other.
5. Cover your face while coughing or sneezing.
6. Disinfect the surfaces used repeatedly (doorknobs, tables, and mobile phones).
7. Avoid crowded areas.
8. Isolate yourself if sick or at greater risk.

## 11. Aerosol Generating Procedures

**Figure 7:**
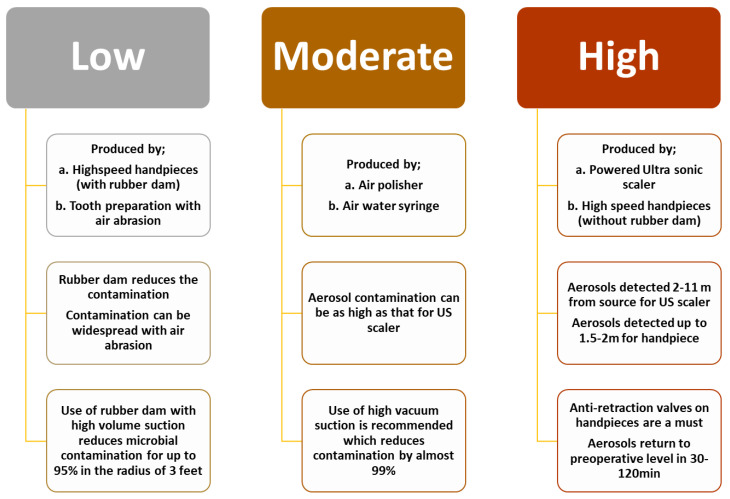
Aerosol-generating dental procedures and associated factors [135].

The CDC says that aerosol generating procedures (Table 8 and Figure 7) are the medical procedures that produce greater concentrations of infective respiratory aerosols than sneezing, coughing, breathing, or speaking [130,131].

Aerosol-generating procedures are hazardous because they release micro-droplets into the air through spray generating equipment [136]. Severe and potentially life-threatening diseases are spread by droplets and aerosols, including pneumonic plague, tuberculosis, influenza, Legionnaire’s diseases, and SARS infections [137]. These droplets may remain in the air or travel long distances and may lead to inhaled infection (Table 9) [138]. Dental practitioners need to develop an understanding of the following [139,140]:

1. Risks associated with different modes of transmission (i.e., droplets, aerosols, and fomites).
2. The sources, nature, kinetics, and the quantity of microbial load in such aerosols.
3. The efficacy of current and emerging practices in mitigating aerosol-generated microbial load.

The coolant used with rotary handpieces and powered scalers has a flow rate of 10 to 40 mL per minute [141] which is 5- to 10-fold greater than unstimulated and stimulated saliva. The dilution of salivary or respiratory pathogens occurs in these settings, reducing the overall pathogenic microbial load and the infectivity of such aerosols. The facial pathologies and fractures management are of greater risk because of the viral load in the oral, nasal, and oropharyngeal mucosa. To reduce the risk of potential infections, we have to adopt principles of simplification of surgery, avoid complicated surgical procedures, and limit the operating times [142,143] as well as implement the various protocols listed in Table 10.

1. Cataloguing of suspicious or high-risk patients (history of fever, respiratory problems, travel history, and contact with a COVID-19 patient during the past 14 days) [143].
2. Repetition of triage [144].
3. Preoperative testing after 48 h, i.e., two RT-PCR tests 24 h apart with a sensitivity of at least 71% [145] (if both tests are not positive, perform surgery with improved airborne protections) [144].
4. Accommodation of patient in an isolated ward or room [144].
5. Execution pace of the preoperative preparation [143,144].

According to the guidelines for performing oral and maxillofacial surgery during COVID-19 by the Association of Osteosynthesis: Cranio-Maxillofacial (AO CMF):

The therapeutic and surgical procedure for facial fractures as suggested by AO CMF are as follows [144,147,151]:

1. Scalpel use over monopolar cautery for skin/mucosal incision.
2. Avoid intra-oral incision, repeated suctioning, and irrigation.
3. Elective surgery must be delayed for non-critical cancer patients unless it does not affect the prognosis.
4. Substitution of power saw by a low-speed drill or osteotome.
5. Application of a low power bipolar cautery for haemostasis.

The following Table 11 [157] highlights the international guidelines for COVID-19 issued by the professional organizations for maxillofacial guidelines [150], the United Kingdom National Health Service [9], the Australian Society for Otolaryngology Head & Neck Surgery, and British Association of Oral and Maxillofacial Surgeons [145].

## 12. Association of Rhino-Cerebral Fungal Infections with COVID-19

Recent literature is showing an association of black fungus (fungal ball) of the lungs and maxillary sinuses with COVID-19 patients. Song et al. have reported an association of COVID-19 with *aspergillus flavus, candida albicans,* and *candida glabrata*. The overall incidence of invasive fungal infections along with COVID-19 co-infection was around 5% [158]. Rabaglaiti et al. studied the corelation of invasive mould infection in established COVID-19 patients and found the overall incidence to be around 11% with a mortality of around 30% in these patients [159]. Waizal-Haiat et al. have reported a case of fatal rhino-orbital mucormycosis along with diabetic ketoacidosis in a COVID-19 patient [160]. Mucormycosis, when it affects the maxillary sinus and orbit, usually presents as pain and swelling in the midface region, involving the eyelids and the nasal fold area. There can be associated paraesthesia of the involved infra-orbital nerve. Other signs and symptoms that can point towards rhino-cerebral mucormycosis can be mobile teeth in the maxilla with discharging sinuses; a presentation very similar to chronic osteomyelitis of the maxilla; black, necrosed palate; nasal blockage; and decreased visual acuity from the affected eye. CT scan or MRI is usually the gold standard for diagnosing involvement of the maxillary and or ethmoidal sinuses. There can be an associated epiphora due to blockage of the nasolacrimal apparatus by the fungus [161]. Management usually includes aggressive anti-fungal treatment through surgical debridement, management of the underlying immunosuppressing state, and supportive therapy to improve the nutritional status of the patients. However, despite all these aggressive treatment modalities, the mortality rate is high, and the disease usually carries a poor prognosis [162].

## 13. Conclusions

Despite scientific advancements, we are still unable to contain the spread of COVID-19, and scientists have yet to develop a definitive treatment for this disease. Currently, there is no evidence that dental healthcare professionals are at a higher risk of airborne viral disease transmission than the general population. Epidemiologic evidence of the prevalence of infections in dental healthcare providers and a comparison to populations as a whole may shine a light on highly protective infection control practices that can be implemented to keep practitioners and patients as safe as possible. Dental professionals should educate patients about the significance of good oral hygiene. Poor oral hygiene is associated with increased plaque deposits and bacterial load, which may lead to bacterial superinfection and risk of complications in COVID-19 patients. Our only preventative measure right now is vaccination and maintenance of cross-infection protocols which can be achieved through proper education of health care workers, patients, and the general public. The COVID-19 pandemic has given us an important message that even the superpowers can collapse and that the most intelligent nations can be startled. Mere weapons cannot defend us and the number of produced medicines cannot suffice our needs. Under the disguise of scientific revolution and industrialization, we have disrupted nature’s equilibrium. A critical question arises: will we realize the importance of our green planet in the post-COVID world?

## Figures and Tables

**Table 1:** Signs and symptoms of COVID-19.

Most Common	Less Common	Most Dangerous
Pyrexia Fatigue Dry cough	Anosmia and ageusia [22,23] Headache [24] Sore throat Diarrhoea Conjunctivitis Skin rash Fingers and toes discoloration	Dyspnoea Chest pain/pressure Loss of movement/speech Heart attack Epilepsy [24] Blood coagulation [25] Cerebral infarction [26] Kidney failure Disseminated intravascular coagulation Acute respiratory distress syndrome and multiple organ failure because of cytokine storm

**Table 2:** Showing relationship of COVID-19 with various comorbid conditions [29].

Hypertension (55.4%) Diabetes (37.3%) Hyperlipidaemia (18.5%) Coronary artery disease (12.4%) Renal disease (11%) Dementia (9.1%) Chronic obstructive pulmonary disease (8.3%) Cancer (8.1%) Atrial fibrillation (7.1%) Heart failure (7.1%)

**Table 3:** Incubation period and onset of symptoms of SARS-CoV-2. Reprinted with permission from Ref. [28]. Copyright 2021 Copyright Elsevier.

	Clinical Features	Population Experiencing (%)
**1 to 3 day(s)** **Onset of symptoms**	Fever generally appears on the first day Cough, sore throat may appear by 3 days	80% of patients get these mild symptoms
**4 to 9 days** **In the lungs**	The virus may reach lungs between 3 to 4 days Difficult breathing may start by 4 to 9 days Acute respiratory distress syndrome due to lungs inflammation between 18 to 25 days	14% of those infected experience these severe symptoms
**8 to 15 days** **In the blood**	From the lungs, the infection may enter the blood By the end of 1 week, sepsis may develop	5% of those infected need admissions to an intensive care unit

**Table 4:** Commonly used drugs for the treatment of COVID-19.

Name of Drug	Potential Role in COVID-19	Problems/Issues/Remarks
**β-D-N4-hydroxycytidine (NHC) [54]**	Ribonucleoside analogue with broad-spectrum antiviral activity (oral route) Effective against Remdesivir-resistant virus, MERS-CoV, SARS-CoV-2, and SARS-CoV in primary HAE cell cultures Reduces virus titres in a dose-dependent manner	Coronavirus may achieve 2-fold resistance after 30 passages [55].
**Interferons (IFN-I and III) [56]**	Produces innate immune response in human cells and stimulates IFN-stimulated genes (ISGs) through JAK/STAT pathway, affecting viral replication at all stages of its replicative cycle. Early administration can decrease the viral spread and can produce extended-lasting responses without inflammatory side effects.	Virus adapts to IFNs by turning over interferon receptors, leading to a diminished response by helper T cells and NK cells. IFNs can produce flu-like symptoms on their own [57,58].
**Chloroquine (CQ), Hydroxychloroquine (HCQ) [56]**	Inhibits intracellular replication of viral particles. It prevents the interaction between the virus and its receptor, thus blocking its effect. Both drugs are immunomodulatory and downregulate Toll-like receptors, thus suppressing the cytokine storm.	HCQ is a less toxic derivative of CQ; hence it is favoured in the treatment of COVID-19. Both these drugs produce reactive oxygen species, which can damage host cells.
**Azithromycin (AZM) [56,59,60]**	Inhibits replication of virus in bronchial cells by decreasing the synthesis of adhesion molecules like ICAM-1. Downregulates cytokine production (IL2, 6, 8), maintains alveolar cell integrity and reduces lung fibrosis Acts synergistically with HCQ in reducing viral load It also prevents bacterial co-infection by Prevotella, which can enhance the pathogenicity of SARS-CoV-2 by internalizing it.	It can cause gastrointestinal upset, nausea, headache, hepatotoxicity, and bacterial resistance. It can prolong QTc interval, ventricular tachycardia, and sudden cardiac arrest by causing intracellular sodium overdose.
**Tocilizumab [61,62]**	Recombinant humanized anti-IL-6 receptor monoclonal antibody, which is a competitive blocker of membrane-bound and soluble IL-6. Potential role in patients presenting with symptoms associated with cytokine storm.	Compochiaro et al. found no statistically significant survival benefit with a slightly increased propensity towards the development of fungal infections; however, it may reduce the need for ventilatory support in hospitalized COVID-19 patients [63].
**Steroids [64,65,66]**	Usually administered steroids include methylprednisolone (32 mg/day), dexamethasone (6 mg/day), and hydrocortisone. Dexamethasone is favoured as it causes minimal fluid retention. It may have a role in reducing the tissue injury due to cytokine storm.	Conflicting body of evidence regarding improvement in survival and decreased hospital stay. May be beneficial but should not be given to all the patients. It can lead towards the development of hyperglycaemia, hypernatremia and mucormycosis and aspergillosis. It can reduce the duration of fever but has no overall effect on the duration of hospitalization.
**Remdesvir [67,68]**	Broad-spectrum antiviral which is an inhibitor of viral RNA-dependent RNA polymerase	Conflicting data on improvement in symptoms with no significant impact on mortality, however, may offer a survival benefit if given early in mild to moderately ill COVID-19 patients.
**Vitamins**	A high dose of vitamin C can prevent cytokine storm in COVID-19 patients, which reduces lung injury and inflammatory damage.	

**Table 5:** Sources, respective companies, and approval status of various COVID-19 vaccines.

Company	Type	Doses	Route	Efficacy	Storage	Approval/Development	Mechanism of Action
**Pfizer–BioNTech**	Nucleoside modified mRNA (BNT162a1 and BNT162b2)	2 shots 21 days apart	I.M inj.	95%	−70 °C	UK approved	Spike proteins and RBD fragments are introduced into the body producing the desired immune response [71].
**Oxford–AstraZeneca**	Viral vector (genetically altered nonreplicating chimpanzee adenovirus)	2 shots 4 to 12 weeks apart	I.M inj.	70%	Regular fridge temperature	UK approved	Specifically deliver genes to the target cells thus providing a trigger to cytotoxic T-cells resulting in killing of infected cells [69].
**Moderna**	Based on lipid nanoparticle-encapsulated mRNA	2 shots 28 days apart	I.M inj.	94.1%	−20 °C	UK approved	Encodes stable form of spike protein of SARS-CoV-2 and educates CD4+ immune cells of the body [72].
**Novavax (NVX-CoV2373)**	Full-length S (spike) Protein-based	2	I.M inj.		Regular fridge temperature	Pending	Promotes migration of leukocytes into lymph nodes thus increasing T-cell, B-cell, and NK cell response [69].
**Janssen (Johnson & Johnson’s)**	Viral vector based using adenovirus or pox virus	1	I.M inj.	66.3%	Regular fridge temperature	Pending	DNA of the adenovirus is modified which helps the body to develop humoral and T-cell based cellular immune response against COVID-19 [73].
**CoronaVac (Sinopharm/Sinovac) (BBIBP-CorV)**	Inactivated virus vaccine		I.M inj.	79%	Regular fridge temperature	Approved by China, Singapore, Saudi Arabia, and Pakistan	Contains virus has been inactivated through UV light/chemicals and elicits antigen-specific antibody response producing plasma cells, T-cells, and memory B-cells [74].
**CanSino Bioloics (Ad5-nCoV) Convidecia**	Non-replicating adenovirus based vaccine	1	I.M inj.	66% to 91%	Regular fridge temperature	Approved by Hungary, China, Mexico, and Pakistan	RBD and spike proteins produce T cell response conferring immunity against virus [69].
**Sputnik V**	Using two non-replicating adenovirus based vector (Ad26, Ad5)	2 doses 21 days apart		Undergoing phase 3 trials		Gamaleya Institute, Moscow.	Dose 1 injects Ad26, and in dose 2 Ad5 is given. This produces an enhanced immune response [69].
**KBP-201 (NCT04473690)**	Protein (RBD-based) subunit vaccine	2 doses 21 days apart	I.M inj.	Currently undergoing phase II trials	-	Pending	RBD in the spike protein binds to ACE-2 receptor producing neutralizing monoclonal antibodies towards SARS-CoV-2 [75].
**Covaxin**	Inactivated virus vaccine					Currently undergoing trials in India	Same as mentioned above under CoronaVac (sinopharm)
**BHPIV3/SARS-S**	Live attenuated virus vaccine	1	I.M. Inj	Currently undergoing phase 2 animal trials		Currently undergoing trials in India and China	Induces production of SARS-CoV neutralizing serum antibodies [69].
**DelNS1-SARS-CoV2-RBD**	Live attenuated vaccine with deletion of NS1 influenza strain	1	Intra-nasal	Currently undergoing phase 2 animal trials			Modified to include SARS-CoV-2 spike protein and is considered more immunogenic than other LAVs [69].
**LUNAR-COV19**	Lipid enabled and unlocked nucleomonomer agent-modified RNA (LUNAR)	1		Currently undergoing phase 1 and 2 trials		Biospace, Singapore	Entry into host cells and mRNA is translated into protiein, s leading to the production of the immune response against SARS-CoV-2 [69].

I.M inj. (Intramuscular injection).

**Table 6:** Prevention and control of COVID-19 during dental healthcare.

**1—Teledentistry and Triage Protocols**	(a) Teledentistry involves using the telephone, SMS, WhatsApp, Skype, Facebook Messenger, Zoom, Microsoft Teams, or emails [108]. (b) Ascertain identity and medical history of the patients along with confirmation of COVID status. (c) Focus on providing appropriate advice, analgesics, and antimicrobials (*the three A’s*) [109]. (d) Determine the urgency of treatment and defer non-emergency treatment. (e) Limit the number of visitors accompanying the patient. (f) Patients and visitors should wear masks [110].
**2—Screening Zone**	(a) Provide face masks and monitor temperature, preferably with a contact-free thermometer. (b) Cough etiquette and hand hygiene instructions. (c) Sanitizers (ABHR) with at least 60% alcohol. (d) If the patient is in emergency conditions for COVID-19, refer him/her to a medical facility and avoid all sorts of dental treatment in a confirmed positive case [111].
**3—Waiting Area**	(a) The waiting area should be well ventilated, and chairs should be at least 2 m apart. (b) Remove frequently touched objects (magazines, etc.). (c) Hand sanitizer should be available. (d) Schedule appointments to minimize patient load [112,113].
**4—Donning Zone**	Clean area PPE wearing sequence including the hand disinfection: Put on gloves in case of double gloving. Wearing covers of shoe, gown, mask/respirator, eye protection and head cap. Perform hand disinfection [114].
**5—Doffing Zone**	Dirty area PPE removing sequence: Remove outer gloves in case double gloving, shoes cover, head cap, gown, inner gloves. Perform hand hygiene (for at least 20 s, use ABHR with alcohol (60%) or wash hands with soap). Remove eye wear and mask/respirator [115].
**6—Dental Surgery Room for Aerosol Generating Procedures**	A closed room. Patients should wear a gown and protective eyewear. Furniture and other non-essential items should be removed. All materials, instruments, paper records, etc., should be outside the surgery. During aerosol procedures, 1 or 2 small openings into the dental surgery, for air inflow, and passing of materials into the surgery. Door should possess a self-closing device [116].
**7—Procedure Infection Control**	Adequate ventilation [117] Masks favoured for dental procedures with aerosol generation are FFP2/N95, FFP3/N95, and NI00 [101]. Valved expirators filter the entering air but releases the unfiltered expired air. Thus, they must be covered with a surgical mask [97,118]. Non-valved expirators are favoured more because they filter both inspired and expired air. Rubber dam isolation. Aseptically set up the instrument tray and the required materials before the procedure. Apply protective barriers. Preprocedural rinse with mouth wash containing 1% hydrogen peroxide for 1 min or 0.2% povidone-iodine for 30 s or chlorhexidine gluconate may reduce the microbial contamination. HV suction with an 8 mm wide suction tip should be held 6–15 mm from an aerosol producing device [119]. Ideally high-speed rotary instruments and handpieces must be avoided. If necessary to use, such instruments must be fitted with anti-retraction system [118].
**8—Dental Surgery Disinfection**	Procedure must be scheduled at the end of day. The clinic must not be accessed for at least 180 min following the procedure. The waste produced by the patient’s treatment with suspected/confirmed COVID-19 is comprehended to be infectious; therefore, yellow coloured double-layer clinical waste bags and “gooseneck” ligation should be used. On the next day or after at least after 180 min, the entire dental surgery should be disinfected meticulously [120].
**9—Dental Surgery Ventilation**	a In-line exhausts with ducts. b Through the wall exhausts. c HVAC systems with HEPA filters can be used for disinfection. The dental clinic might be converted to a negative pressure room, with >12 air changes an hour [116].
**10—Dental Equipment Maintenance**	Follow the guidelines of IFU for the maintenance of dental unit water-lines, autoclave, compressors, radiography equipment, and suctions [116].

**Table 7:** Various emergency and non-emergency dental procedures according to COVID-19 guidelines [125].

Dental Non-Emergency Procedures	Dental Emergency Procedures
New/periodic oral examinations	Uncontrolled bleeding
Routine x-rays	Cellulitis/bacterial facial space infection
Routine dental cleaning as well as preventive therapies	Severe dental pain (pulpitis)
Extraction of asymptomatic teeth	Pericoronitis/3rd molar pain
Restorative dental procedures (fillings, crowning)	Dry socket
Recall/revisit	Tooth fracture
	Dento-alveolar trauma
	Painful broken filling
	Adjustment of ortho-wire damaging gums
	Post-surgery treatment

**Table 8:** Aerosol-generating medical procedures [132,133,134].

1. Tracheostomy and tracheal intubation procedures	2. Positive-pressure mechanical ventilation and CPAP
3. Bronchoscopy	4. Intubation and extubation procedures
5. Surgery, autopsy, or post-mortem procedures with high-speed devices	6. High frequency oscillatory ventilation
7. Cardiopulmonary resuscitation	8. High-flow oxygen therapy
9. Sputum induction	10. Airway suctioning
11. FEES and VFSS	12. Nebulized or aerosol therapy
CPAP (Continuous positive airway pressure); FEES (Fibreoptic endoscopic evaluation of swallowing); VFSS (Video fluoroscopic swallowing study)	

**Table 9:** Showing different types of aerosols and droplets and their significance [141].

Droplet Type	Description
Splatter droplets	Particle size ≥ 50 µm, briefly airborne, and spread by close contact (typically within 1 m) with the host.
Aerosols	Particle size < 50 µm, carry viable pathogens, remain airborne for prolonged period, and spread to distant surfaces.
Droplets > 5 µm	Remain in the upper respiratory tract.
Droplets ≤ 5 µm	Might be inhaled into the lower respiratory tract.
Droplets ≤ 1 µm	Can enter alveoli.

**Table 10:** Protocols for maxillofacial procedures during COVID-19 pandemic.

Pre-Procedure Protocols	During Procedure Protocols	Post-Procedure Protocols
Medical and dental history, physical examination, and auxiliary tests should be conducted. Povidone (0.2%) or hydrogen peroxide (1%) containing mouthwashes efficiently lowers the droplets and aerosols number formed throughout oral operations [9,146,147,148].	PPE: eye protection, masks (N95 or FFP2), surgical gloves, and fluid-resistant gown. Limit the instruments and materials used during the procedure [149]. Surgical procedures of positive patients should be carried out in a negative pressure room [150]. However, COVID-19 negative patients can be in non-negative pressure rooms [151]. Use of PAPR is strongly recommended [152,153,154]. Allocated protection factor for PAPR is 25–100, while for N95 it is 10 [155]. All staff except the anaesthesia team should remain outside the operating room 10 min following intubation and extubation [156].	Allocate patients in separate rooms. Periodic control of hypertension, temperature, oxygen saturation, and heart rate. Monitoring devices should be different for each patient. Medical treatment decreasing the use of glucocorticoids. Early patient mobilization to reduce hospitalization [143].
personal protective equipment (PPE); powered air purifying respirator (PAPR)		

**Table 11:** International guidelines for management of cranio-maxillo-facial trauma [157].

**Urgency for operation**	Emergent (require surgical intervention in ≤24 h)	Urgent (require surgical intervention for bone union)
**Patient presentation**	Compromised airway or vision, uncontrolled bleeding, or combined intracranial or upper facial fracture	Facial fracture causing functional or cosmetic deformity including displaced cranio-orbital fractures, orbital dystopia, and naso-orbito-ethmoid fractures
**COVID-19 screening**	RT-PCR or rapid COVID test	RT-PCR or rapid COVID-19 test

## Data Availability

Data sharing not applicable.
